# Molecular characterization of invasive meningococcal isolates in Burkina Faso as the relative importance of serogroups X and W increases, 2008–2012

**DOI:** 10.1186/s12879-018-3247-x

**Published:** 2018-07-18

**Authors:** Adam C. Retchless, Malika Congo-Ouédraogo, Dinanibè Kambiré, Jeni Vuong, Alex Chen, Fang Hu, Absetou Ky Ba, Abdoul-Salam Ouédraogo, Soumeya Hema-Ouangraoua, Jaymin C. Patel, Rasmata Ouédraogo Traoré, Lassana Sangaré, Xin Wang

**Affiliations:** 1 Meningitis and Vaccine Preventable Diseases Branch, Division of Bacterial Diseases, National Center for Immunization and Respiratory Diseases, Centers for Disease Control and Prevention, Atlanta, USA; 2 Centre Hospitalier Universitaire Yalgado Ouédraogo, Ouagadougou, Burkina Faso; 3 Centre Hospitalier Universitaire Pédiatrique Charles de Gaulle, Ouagadougou, Burkina Faso; 4 Laboratoire National de Santé Public, Ouagadougou, Burkina Faso; 5 Centre Hospitalier Universitaire Sanou Sourou, Bobo-Dioulasso, Burkina Faso; 6 Centre Muraz, Bobo-Dioulasso, Burkina Faso; 7 Epidemic Intelligence Service, Centers for Disease Control and Prevention, Atlanta, USA

**Keywords:** *Neisseria meningitidis*, Burkina Faso, Africa, Epidemic, Surveillance

## Abstract

**Background:**

*Neisseria meningitidis* serogroup A disease in Burkina Faso has greatly decreased following introduction of a meningococcal A conjugate vaccine in 2010, yet other serogroups continue to pose a risk of life-threatening disease. Capsule switching among epidemic-associated serogroup A *N. meningitidis* strains could allow these lineages to persist despite vaccination. The introduction of new strains at the national or sub-national levels could affect the epidemiology of disease.

**Methods:**

Isolates collected from invasive meningococcal disease in Burkina Faso between 2008 and 2012 were characterized by serogrouping and molecular typing. Genome sequences from a subset of isolates were used to infer phylogenetic relationships.

**Results:**

The ST-5 clonal complex (CC5) was identified only among serogroup A isolates, which were rare after 2010. CC181 and CC11 were the most common clonal complexes after 2010, having serogroup X and W isolates, respectively. Whole-genome phylogenetic analysis showed that the CC181 isolates collected during and after the epidemic of 2010 formed a single clade that was closely related to isolates collected in Niger during 2005 and Burkina Faso during 2007. Geographic population structure was identified among the CC181 isolates, where pairs of isolates collected from the same region of Burkina Faso within a single year had less phylogenetic diversity than the CC181 isolate collection as a whole. However, the reduction of phylogenetic diversity within a region did not extend across multiple years. Instead, CC181 isolates collected during the same year had lower than average diversity, even when collected from different regions, indicating geographic mixing of strains across years. The CC11 isolates were primarily collected during the epidemic of 2012, with sparse sampling during 2011. These isolates belong to a clade that includes previously described isolates collected in Burkina Faso, Mali, and Niger from 2011 to 2015. Similar to CC181, reduced phylogenetic diversity was observed among CC11 isolate pairs collected from the same regions during a single year.

**Conclusions:**

The population of disease-associated *N. meningitidis* strains within Burkina Faso was highly dynamic between 2008 and 2012, reflecting both vaccine-imposed selection against serogroup A strains and potentially complex clonal waves of serogroup X and serogroup W strains.

**Electronic supplementary material:**

The online version of this article (10.1186/s12879-018-3247-x) contains supplementary material, which is available to authorized users.

## Background

In December 2010, Burkina Faso initiated a mass vaccination campaign to fully immunize its population between the ages of 1–29 with a novel polysaccharide-tetanus toxoid conjugate vaccine against serogroup A *Neisseria meningitidis* (PsA-TT) [1]. The 10-day vaccination campaign vaccinated approximately 11 million people, achieving 96% coverage among the target population. In parallel with the vaccination campaign, Burkina Faso expanded its case-based meningitis surveillance program and laboratory capacity to evaluate the long-term effectiveness of PsA-TT vaccination. Surveillance data identified a 99.8% reduction in the risk of meningococcal A meningitis [2], while carriage studies reported a corresponding decrease in carriage of *N. meningitidis* serogroup A (NmA) [3]. Similar success has been noted in several other countries within the African “meningitis belt”, which stretches from Senegal in the west to Ethiopia in the east [4].

Despite the effectiveness of PsA-TT in reducing disease due to NmA, other serogroups continue to present a risk for meningococcal disease in Burkina Faso and in the African meningitis belt [1, 4–6]. Burkina Faso was struck by epidemics of serogroup X (NmX) disease in 2010 [7] and serogroup W (NmW) disease in 2012 [8]. Multiple strategies are being considered to develop vaccines that protect against NmW and NmX disease in the meningitis belt. While the serogroup W polysaccharide is an established vaccine component [8], the utility of serogroup X polysaccharide is under investigation [9]. The use of protein antigens (FHbp, NadA, NhbA) was pioneered for protection against disease caused by NmB strains [10], but is also being examined for protection against NmW and NmX strains [11, 12].

Multilocus Sequence Typing (MLST) [13] assigned the NmX isolates to the sequence type 181 clonal complex (CC181) and the NmW isolates to CC11. Immediately prior to the introduction of the PsA-TT vaccine, the primary lineage of NmA in Burkina Faso was CC5. While vaccination against serogroup A disease is expected to reduce the frequency of disease due to CC5, the acquisition of capsular synthesis genes from other *N. meningitidis* strains could produce CC5 variants against which PsA-TT provides no protection [14, 15]. One instance of a CC5 NmA strain converting to NmX has been documented in China [16], illustrating the possibility of capsular switching in this lineage, while also demonstrating that vaccine escape is not sufficient for a strain to cause high rates of disease [17].

Meningococcal populations in meningitis belt communities have been observed to exhibit “clonal waves”, where previously unobserved strains of *N. meningitidis* show rapid increases in rates of carriage and disease, and then become undetectable in both disease surveillance and carriage studies after a few years [18]. While a decade-long longitudinal study has described the genetic diversity of three successive clonal waves of NmA in a single community [19, 20], the geographic scale of clonal wave dynamics is still unclear. At one extreme, clonal waves could be largely localized, with minimal dispersal of *N. meningitidis* between human communities during a wave; at the other extreme, the clonal wave could involve a nation-wide population of *N. meningitidis* with frequent transmission between human communities. Phylogenetic analysis based on whole genome sequence data is capable of distinguishing geographic subpopulations of *N. meningitidis* during nationwide epidemics in Burkina Faso [21], but evaluating the stability of geographic subpopulations requires geographically diverse, multi-year strain collections.

The NmX and NmW outbreaks in Burkina Faso were each preceded by low rates of carriage and disease for the respective serogroups [2, 3], and clonal waves could have been initiated by introduction of a strain from another country in the meningitis belt, where both CC181 NmX and CC11 NmW have been detected since the 1990s [21, 22]. CC181 NmX isolates from Africa collected prior to 2010 fall into two phylogenetic groups [22]. Meanwhile, CC11 NmW isolates from Africa belong to several subclades within a globally distributed CC11 NmW clade [21, 23]. The CC11 NmW isolates collected in Burkina Faso during 2011 and 2012 have been shown to descend from the strain identified during the Hajj-related outbreak of 2000, which also included isolates collected in Mali during 2012 and Niger during 2015 [5, 21].

Here, we describe a convenience sample of isolates collected from cases of invasive meningococcal disease by the Burkina Faso national surveillance system between 2008 and 2012. MLST and serogrouping were performed to assess the frequency of capsular switching among CC5 isolates, and show occurrence of clonal waves. To explore the stability of geographic subpopulations during clonal waves of *N. meningitidis*, we applied a spatiotemporal analysis to the phylogenetic relationships among NmX isolates collected during and after the 2010 epidemics, as well as among the NmW isolates collected before and during the 2012 epidemics.

## Methods

### Isolate collection

**Fig. 1:**
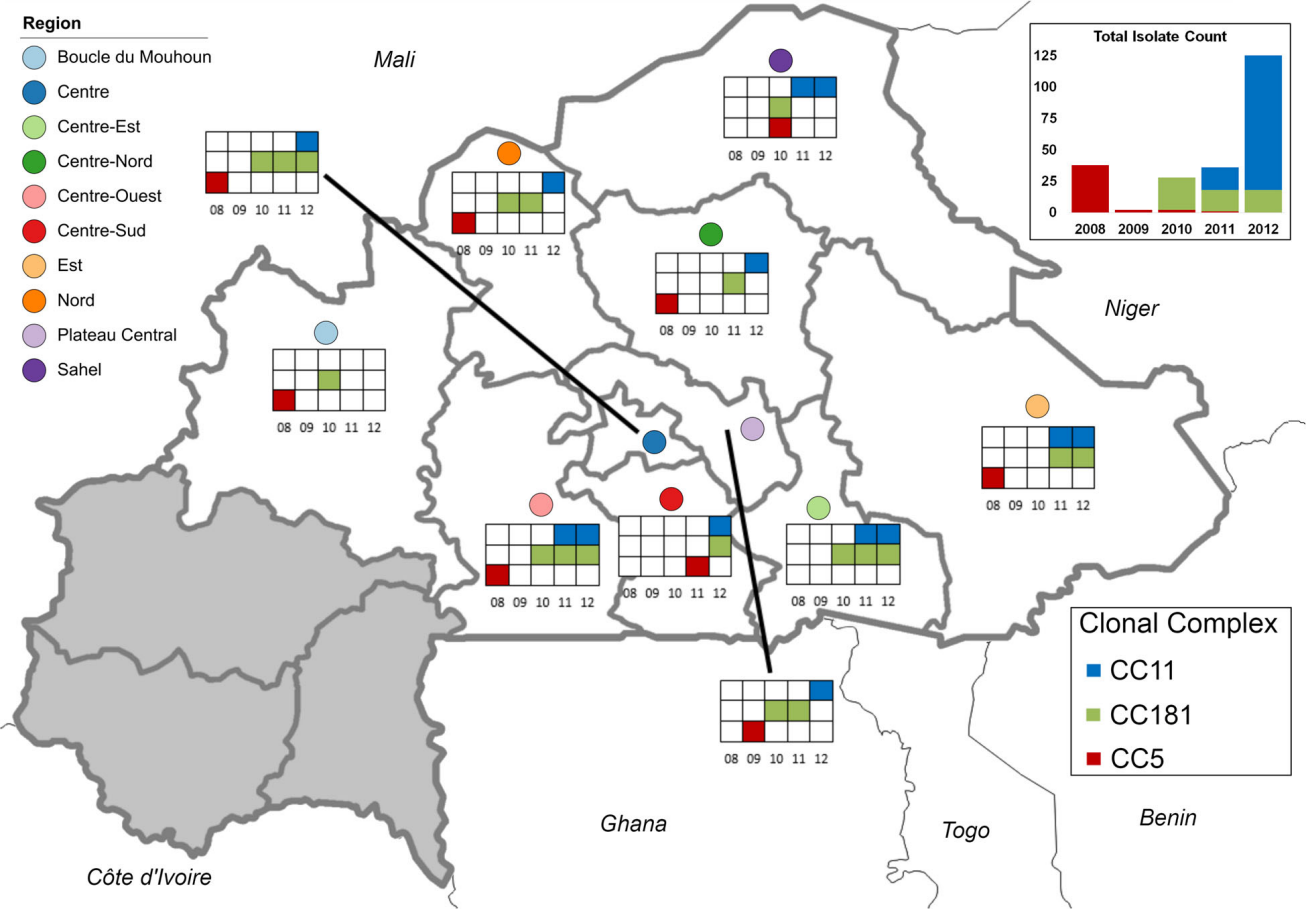
Provenance of isolates belonging to the three major clonal complexes. Colored squares show the years in which isolates were obtained in each region of Burkina Faso. The inset shows the count of isolates from each year. The names of neighboring countries are written in italics

The primary isolate collection (*n* = 236) originated from the Burkina Faso national surveillance network (Table 1). The isolates were collected through convenience sampling and limited to those for which the originating health district was documented. The isolates came from 37 of 63 (59%) health districts in Burkina Faso, representing 10 of 13 administrative regions (77%) (Fig. 1). *N. meningitidis* identification was confirmed using species-specific real-time PCR, while serogroup was identified using slide agglutination and confirmed using real-time PCR [24].

**Table 1:** Molecular typing of meningococcal isolates according to year, grouped by serogroup and clonal complex

	2008	2009	2010	2011	2012	Total
NmA, CC5 (total)^a^	**38**	**2**	**2**	**1**		**43**
P1.20,9: F3–1: ST-2859^b^	36	1	2	1		40
P1.20,9: F3–1: ST-7		1				1
P1.20,9: F3–1: ST-9752	2					2
NmW, CC11 (total)				**18**	**110**	**128**
P1.5,2: F1–1: ST-11				17	106	124
P1.5,2: F6–3: ST-11					1	
P1.5,2: F1–1: ST-2961				1		1
P1.5,2: F1–1: ST-9766					3	3
NmW, CC175 (total)			**1**		**2**	**3**
P1.5–1,2–36: F5–1: ST-8638			1			1
P1.5–1,2–36: F5–1: ST-9357					1	1
P1.5–1,2: F5–1: ST-9357					1	1
NmX, CC181 (total)			**26**	**17**	**18**	**61**
P1.5–1,10–1: F1–31: ST-181			24	10	18	52
P1.5–1,10–1: F5–69: ST-181			2	7		9
NmY, CC23 (total)					**1**	**1**
P1.5–1,2–2: F5–8: ST-4375					1	1
Total	**38**	**2**	**29**	**36**	**131**	**236**

^a^Numbers in bold are total counts for each group

^b^Molecular typing results are PorA type, FetA type, and Sequence Type

**Fig. 2:**
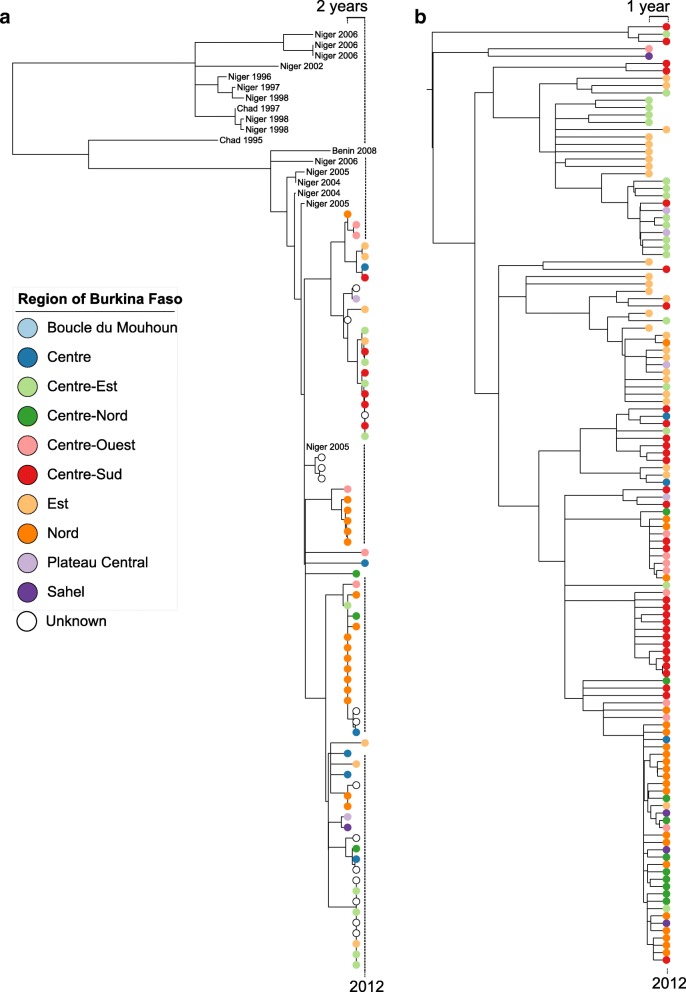
Phylogenetic relationship among isolates collected in different regions of Burkina Faso according to year. **a** CC181 NmX collected 2010–2012. **b** CC11 NmW isolates collected 2011–2012. Colored circles indicate the region of Burkina Faso in which the isolate was collected. Additional isolates are labeled with country and year of origin, except for isolates from Burkina Faso, which are indicated with a non-filled circle. The year of isolation is indicated by the position on the horizontal axis. The location of isolates from 2012 is marked at the bottom, and the range of years represented in the primary isolate collection is marked at the top. A dashed vertical line is included as a guide, indicating the location of 2012

An additional 20 NmX isolates were included to provide phylogenetic context (Fig. 2). These were obtained either from the former WHO Collaborating Centre in Marseille, or from Burkina Faso laboratories without documentation of the originating health district.

### Genome sequencing

Draft genome sequences were generated for 193 isolates from 250 base pair (bp), paired-end read data generated by an Illumina HiSeq 2500 (CDC Biotechnology Core Facility) as previously described [25]. Improved assemblies were generated for 11 isolates using a Pacific Biosciences (PacBio) RSII sequencer with P4-C2 sequencing chemistry from 10 kilobase (kb) libraries created with DNA Template Prep Kit 3.0 and DNA/Polymerase Binding Kit P6 v2. Reads were assembled using PacBio’s Hierarchical Genome Assembly Process v3 (HGAP) (Chin, Nat Methods 2013) where 30 megabases of the longest corrected reads were used for the initial assembly. Sequences were established to be complete circular DNA molecules by identifying repeats at the ends of the single contig, removing the repeat from one end, transferring sequence from the 3′ to the 5′ end, and confirming that the manual join point was supported by remapped reads. Closed contigs were then reoriented so that the first 5 kb aligned with the beginning of the FAM18 reference genome (GenBank Accession: AM421808.1). Genome sequences were submitted to NCBI under BioProject PRJNA338313.

### Molecular typing

For isolates with available whole genome sequence data, peptides and MLST alleles were identified based on a BLAST search of the assembled genomes against the PubMLST allele lists [13]. For isolates without whole genome sequence data, loci were sequenced and interpreted as described by Jolley et al. [26] and Wang et al. [27]. NadA was categorized by the convention of variant and peptide identifier [28], while NhbA and FHbp were identified by PubMLST peptide identifiers.

### Phylogenetic analysis

Previously published genomes for the *N. meningitidis* ST-11 and ST-181 clonal complexes were downloaded from the PubMLST web server on April 28, 2016 [13]. A preliminary phylogenetic tree for the 1052 CC11 NmW isolates was constructed using RAxML v8.2.4 [29] based on 13,147 core single nucleotide polymorphisms (SNPs) identified using kSNP3 (k-mer = 25) [30]. All CC11 NmW isolates from this study belonged to the clade previously identified among isolates from Burkina Faso and Mali 2011–2012 (subclade IVa of Retchless et al... [21]); therefore, the final CC11 phylogeny was limited to the isolates from this study and an ancestral isolate from the Hajj-related outbreak strain to use as an outgroup (M07149). The relationship of these isolates to the collection described by Lucidarme et al [23] is shown in Additional file 1.

Genomes were aligned to M07149 (CC11) and M22348 (CC181) by first orienting contigs using the Mauve Contig Mover with then using progressive Mauve (HMM identity = 95%) [31]. The LCBs in the XMFA alignment file were oriented to match the reference sequence, constructing a mask for sites within 5 bases of a gap character or between gaps less than 30 bases apart. LCBs less than 5 kb were masked, as were the 50 positions at the edge of each LCB.

Phylogenetic topology was calculated from the alignment without the masked sites, using phyML with 10 random starting points and 100 bootstrap replicates [32]. The branch lengths of that midpoint-rooted tree were adjusted to reflect the expected number of point mutations using ClonalFrameML to identify recombinant regions, using the full alignment with masked sites [33]. Figures were created by first applying a temporal constraint to the phylogenies using the QPD algorithm within the program LSD version 0.2 [34], then displaying the phylogeny with iToL [35].

The CC181 NmX core alignment of 93 genomes included 1,465,622 nucleotides (67% of M22348) with 9059 polymorphic positions. PhyML estimated kappa (the transition:transversion ratio) as 5.05. ClonalFrameML identified 179 recombination events, estimating parameters R/theta = 0.35 (frequency of recombination events to point mutation events), nu = 4.7% (mean polymorphisms in recombinant tracts), and delta = 801 bp (mean recombination tract length). The CC11 NmW core alignment of 129 genomes included 1,910,183 nucleotides (88% of M07149) with 5228 polymorphic sites. PhyML estimated kappa as 4.35, while ClonalFrameML identified 109 recombination events, estimating parameters R/theta = 0.212, nu = 5.1%, and delta = 621 bp.

### Geographic and temporal analysis of phylogenetic diversity

Geographic and temporal clustering of diversity was evaluated by comparing the mean diversity within and between groups of isolates defined by the region and year in which they were collected (e.g. Nord 2010). Diversity was calculated as the ClonalFrameML tree distance. The probability of obtaining lower or equal diversity estimates (p) from random samples of the isolate collection was calculated by repeating the diversity estimate for 10,000 simulated isolate collections constructed by permuting the assignment of isolates to groups. For evaluation of within-group diversity, individual isolates were randomly assigned to groups, preserving the size of each group. For evaluation of diversity within regions across years, isolates from each region were reassigned as a group to other regions sampled in the same year. Conversely, for evaluation of diversity within years across regions, isolates from each year were reassigned as a group to other years during which the same region was sampled. Data analysis was performed with SciPy (version 0.18; E. Jones, E. Oliphant, P. Peterson, et al. SciPy: Open Source Scientific Tools for Python, 2001 [http://www.scipy.org/]) and BioPython 1.68 [36].

## Results

### Molecular typing

A total of 236 surveillance isolates were evaluated, belonging to four different serogroups and five different clonal complex (Table 1). Each clonal complex included a single serogroup. The isolates originated from 10 regions in the Center/North/East of Burkina Faso; Fig. 1 shows the regions from which CC5, CC181, and CC11 isolates originated each year. CC5 NmA isolates were primarily from 2008, with a few isolates from 2009, 2010, and 2011, and no isolates from 2012. CC181 NmX isolates were collected between 2010 and 2012. The CC181 isolates were collected in seven regions during each of 2010 and 2011, and five during 2012; six regions contributed CC181 isolates across consecutive years. CC11 NmW isolates were collected in four regions in 2011 and nine regions in 2012, with four regions contributing isolates in both years.

Evaluation of PorA, FetA, and Sequence Type (ST) identified several infrequent variants among the isolates (Table 1). The variation among genes targeted by protein-based vaccines was examined for the CC5, CC11, and CC181 isolates. Isolates from CC5 were all identical at these loci, with NadA 2/3.8, NhbA p0126, and FHbp peptide 5 from subfamily B/v1. All 61 isolates from CC181 were missing NadA and had NhbA p0359, while 60 had FHbp peptide 74 and 1 had peptide 932 (both peptides belonging to subfamily B/v1). All 128 isolates from CC11 had NadA 2/3.6 and NhbA p0096, while 116 had FHbp peptide 9 (subfamily B/v1), 12 had FHbp peptide 12 (subfamily A/v2–3), and 1 had FHbp peptide 613 (subfamily B/v1).

### Phylogeny of clonal complex 181

Whole genome sequences were obtained for 56 CC181 NmX isolates collected in Burkina Faso, 2010–2012, and a maximum likelihood phylogenetic analysis was performed to explore geographic and temporal population structure (Fig. 2a). When isolates from other meningitis belt countries were included, two major clades were evident (bootstrap = 100%), as described by Agnememel et al. [22]. One clade contained isolates from Niger (1997–2006) and the other contained isolates from Burkina Faso 2007–2012, with a few isolates collected in Niger since 2004. The monophyly of the isolates from Burkina Faso 2010–2012 could not be established, since the smallest clade that contained all Burkina Faso isolates from 2010 to 2012 also included the isolates from Niger 2005 and Burkina Faso 2007 with weak support (bootstrap = 4%).

To explore the geographic and temporal population dynamics, isolates were categorized according to the year (2010–2012) and region of Burkina Faso in which they were collected. The relatedness across regions and years was then summarized based on the branch lengths within a recombination-adjusted phylogenetic tree. Pairs of isolates collected in the same region of Burkina Faso during a single year were on average more closely related than pairs of isolates in the collection as a whole (1.55 × 10^− 5^ vs. 1.88 × 10^− 5^ mutations per site; *p* = 0.001). When compared across regions, isolates collected during the same year were more closely related than isolates collected across all years (1.65 × 10^− 5^ vs. 1.90 × 10^− 5^ mutations per site; *p* = 0.021). However, when compared across years, isolates collected in the same region were not significantly more closely related to each other than to isolates collected across all regions (1.93 × 10^− 5^ vs. 1.99 × 10^− 5^ mutations per site; *p* = 0.346).

The diversity among isolates collected during 2011 was higher (2.02 × 10^− 5^ substitutions per site) than among isolates collected during 2010 (1.75 × 10^− 5^ substitutions per site) or 2012 (1.12 × 10^− 5^ substitutions per site). This is reflected in the phylogenetic topology, where the isolates from 2012 were largely from a clade that contained only a single isolate from 2010.

### Phylogeny of clonal complex 11

All 128 CC11 NmW isolates from Burkina Faso 2010–2012 belonged to a clade previously identified from Burkina Faso and Mali during 2011–2012 (subclade IVa of Retchless et al. [21]). This clade is separate from the African isolates described by Lucidarme et al. [23], although one isolate collected in France during 2014 belongs to the clade (Additional file 1).

The smallest clade that includes all isolates from 2012 also includes all isolates from 2011 (Fig. 2b). The clustering of isolates within the phylogeny is reflected in the region from which they were collected. Isolates collected in the same region in the same year had lower mean diversity than the collection as a whole (1.57 × 10^− 5^ substitutions per site vs 1.85 × 10^− 5^ substitutions per site; *p* < 0.0001). When compared across regions, isolates collected during the same year were slightly more closely related than isolates collected across all years (1.72 × 10^− 5^ vs. 1.84 × 10^− 5^ mutations per site). For this comparison, a meaningful *p*-value cannot be calculated because the observed diversity (1.72 × 10^− 5^ mutations per site) is the lowest among the 16 possible permutated assignments of isolates to years in the four regions that had isolates in both 2011 and 2012. When compared across years, isolates collected in the same region were slightly more closely related to each other than to isolates collected across all regions (2.07 × 10^− 5^ vs. 2.30 × 10^− 5^ mutations per site; *p* = 0.150).

## Discussion

Following the introduction of PsA-TT to Burkina Faso in 2010, serogroup A disease was greatly reduced [2]. The isolate collection described here reflects this reduction and furthermore provided no indication of capsular switching, since isolates from each clonal complex belonged to a single serogroup (Table 1). Consequently, the abundance of CC5 isolates in the surveillance collection was reduced along with the serogroup A reduction, and no CC5 isolates were identified in 2012. Instead, most of the 193 non-serogroup A isolates belonged to two clonal complexes that have been associated with epidemics in 2010 (CC181, NmX) and 2012 (CC11, NmW). The predominance of these lineages in this isolate collection is consistent with surveillance reports showing that serogroups A, W, and X accounted for the vast majority of meningococcal disease cases in Burkina Faso between 2008 and 2012 [2, 37]. Other clonal complexes are also known to cause disease in Burkina Faso, including CC23 (NmY) and CC175 (NmW) that were identified in this isolate collection, and CC167 (NmY) and CC192 (nongroupable) that were identified among Burkina Faso isolates collected from 2004 to 2010 [38].

Both NmX and NmW disease cases continue to be reported in Burkina Faso and other countries of the meningitis belt [6], with NmC recently emerging as a substantial cause of disease, particularly in Nigeria and Niger [5]. While polysaccharide-based vaccines either exist or are in development for these serogroups, protein-based vaccines may also provide protection against disease if the targeted surface proteins are expressed. The CC181 NmX and CC11 NmW isolates collected from Burkina Faso from 2010 to 2012 all encoded vaccine antigens that are targeted by two commercially produced protein-based serogroup B meningococcal vaccines [10]. The components of these vaccines could form the basis for future serogroup-independent vaccines that could be used in Africa [11].

Both the CC181 NmX and CC11 NmW isolates formed phylogenetic clades with low diversity, yet geographic population structure among the isolates could still be detected based on the phylogenetic relationships that were inferred from whole genome sequence data (Fig. 2). For each clonal complex, isolates collected during the same year in a single region had lower sequence diversity than was measured among the total collection of isolates belonging to that clonal complex (after properly weighting homologous recombination events). The reduced diversity indicates that over short time scales, transmission of *N. meningitidis* is primarily within geographically limited human populations. However, the current analysis cannot discern whether closely related isolates are recovered from cases spread across a region or are only found on smaller scales, such as within sanitary districts or among close contacts. Over multiple years, isolates collected during the same year across different regions were more similar than isolates collected in the same region across different years. This suggests that meningococcal strains readily move between regions of Burkina Faso over the course of a few years. Furthermore, the changes to geographic populations over years suggests that clonal waves may include the successive replacement of strains by close relatives, rather that the establishment of geographically stable multi-year populations. The identification of strain replacement among these invasive *N. meningitidis* strains in Burkina Faso is primarily limited by the irregular geographic distribution of isolates in this collection, which resulted in only a few regions being represented by isolates of the same clonal complex in multiple years. An additional limitation is the short span of years represented by the CC181 NmX and CC11 NmW isolates.

For both CC181 and CC11, the phylogenetic analysis indicated that the strains evaluated here had diverged prior to the first year that they were identified in Burkina Faso [7, 8, 21]. The CC181 isolates collected in Burkina Faso starting in 2010 included two major phylogenetic branches that likely shared a common ancestor before or during 2005, based on the inclusion in that clade of an isolate from Niger collected in 2005. The ancestry of the CC11 isolates is less clear due to the absence of older isolates belonging to the clade. However, the diversity of isolates from 2012 indicates that their most recent common ancestor pre-dated 2011, when the clade was first identified in Burkina Faso. These results indicate that the *N. meningitidis* populations that cause epidemics are likely present for several years in Burkina Faso or neighboring countries, rather than emerging from a single clonal introduction into the country near the onset of the epidemic.

## Conclusions

Following the elimination of serogroup A *N. meningitidis* disease epidemics in Burkina Faso, two recent epidemics in Burkina Faso were caused by pathogen populations exhibiting small amounts of genotypic variation. Whole genome sequencing data included sufficient diversity to identify geographic structure within clonal *N. meningitidis* populations. Sampling of NmX isolates over multiple years indicated mixing of the NmX population between different regions and potential strain replacement within some regions. Expanded surveillance of *N. meningitidis* disease in the African meningitis belt is providing a broad understanding of how *N. meningitidis* epidemiology is changing in response to PsA-TT vaccination. Analysis of the genomic diversity of surveillance isolates obtained by annual, geographically representative collections can elucidate pathogen dissemination at the scale of both countries and continents and generate hypotheses regarding both the genetic and epidemiological contributors to disease risk.

## Additional file

Additional file 1: (PDF) Unrooted phylogeny of international NmW CC11 isolates. The 128 isolates from this study are identified by black squares, the Hajj-related outbreak isolate is identified by a black star, and the remaining 470 isolates are identified according to the categories defined by from Lucidarme et al. [23]. Arrows mark isolates that are not in the same category as the most closely related isolates: the Hajj-related outbreak isolate (M07149) and an “Anglo/French Hajj strain” isolate collected in France during 2014 (M14 240,446). The tree is scaled by the number of parsimonious substitutions per branch, identified by kSNP3. Branches with bootstrap support < 70% have been deleted. (PDF 33 kb)

## Abbreviations

bp: Basepair
CC11: ST-11 clonal complex
CC181: ST-181 clonal complex
CC5: ST-5 clonal complex
CDC: Centers for Disease Control and Prevention
HMM: Hidden Markov Model
MLST: Multilocus Sequence Typing
NmA: *Neisseria meningitidis* serogroup A
NmC: *Neisseria meningitidis* serogroup C
NmW: *Neisseria meningitidis* serogroup W
NmX: *Neisseria meningitidis* serogroup X
PsA-TT: Polysaccharide-tetanus toxoid conjugate vaccine against serogroup A
SNP: Single Nucleotide Polymorphism
ST: Sequence Type

## References

[1] Djingarey MH, Diomande FV, Barry R, Kandolo D, Shirehwa F, Lingani C, Novak RT, Tevi-Benissan C, Perea W, Preziosi MP (2015). Introduction and rollout of a new group a meningococcal conjugate vaccine (PsA-TT) in African Meningitis Belt countries, 2010-2014. Clin Infect Dis.

[2] Novak RT, Kambou JL, Diomande FV, Tarbangdo TF, Ouedraogo-Traore R, Sangare L, Lingani C, Martin SW, Hatcher C, Mayer LW (2012). Serogroup a meningococcal conjugate vaccination in Burkina Faso: analysis of national surveillance data. Lancet Infect Dis.

[3] Kristiansen PA, Ba AK, Ouedraogo AS, Sanou I, Ouedraogo R, Sangare L, Diomande F, Kandolo D, Saga IM, Misegades L (2014). Persistent low carriage of serogroup a *Neisseria meningitidis* two years after mass vaccination with the meningococcal conjugate vaccine, MenAfriVac. BMC Infect Dis.

[4] Trotter CL, Lingani C, Fernandez K, Cooper LV, Bita A, Tevi-Benissan C, Ronveaux O, Preziosi MP, Stuart JM. Impact of MenAfriVac in nine countries of the African meningitis belt, 2010-15: an analysis of surveillance data. Lancet Infect Dis. 2017;17(8):867–72. 10.1016/S1473-3099(17)30301-8 28545721

[5] Kretz CB, Retchless AC, Sidikou F, Issaka B, Ousmane S, Schwartz S, Tate AH, Pana A, Njanpop-Lafourcade BM, Nzeyimana I (2016). Whole-genome characterization of epidemic *Neisseria meningitidis* Serogroup C and resurgence of Serogroup W, Niger, 2015. Emerg Infect Dis.

[6] World Health Organization (2017). Epidemic meningitis control in countries of the African meningitis belt, 2016. Wkly Epidemiol Rec.

[7] Delrieu I, Yaro S, Tamekloe TA, Njanpop-Lafourcade BM, Tall H, Jaillard P, Ouedraogo MS, Badziklou K, Sanou O, Drabo A (2011). Emergence of epidemic *Neisseria meningitidis* serogroup X meningitis in Togo and Burkina Faso. PLoS One.

[8] MacNeil JR, Medah I, Koussoube D, Novak RT, Cohn AC, Diomande FV, Yelbeogo D, Kambou JL, Tarbangdo TF, Ouedraogo-Traore R (2014). *Neisseria meningitidis* serogroup W, Burkina Faso, 2012. Emerg Infect Dis.

[9] Acevedo R, Zayas C, Norheim G, Fernandez S, Cedre B, Aranguren Y, Cuello M, Rodriguez Y, Gonzalez H, Mandiarote A (2017). Outer membrane vesicles extracted from *Neisseria meningitidis* serogroup X for prevention of meningococcal disease in Africa. Pharmacol Res.

[10] Harrison LH (2015). Vaccines for prevention of group B meningococcal disease: not your father's vaccines. Vaccine.

[11] Pajon R, Lujan E, Granoff DM (2016). A meningococcal NOMV-FHbp vaccine for Africa elicits broader serum bactericidal antibody responses against serogroup B and non-B strains than a licensed serogroup B vaccine. Vaccine.

[12] Hong E, Giuliani MM, Deghmane AE, Comanducci M, Brunelli B, Dull P, Pizza M, Taha MK (2013). Could the multicomponent meningococcal serogroup B vaccine (4CMenB) control *Neisseria meningitidis* capsular group X outbreaks in Africa?. Vaccine.

[13] Jolley KA, Maiden MC (2010). BIGSdb: scalable analysis of bacterial genome variation at the population level. BMC Bioinformatics.

[14] Mustapha MM, Marsh JW, Krauland MG, Fernandez JO, de Lemos AP, Dunning Hotopp JC, Wang X, Mayer LW, Lawrence JG, Hiller NL (2016). Genomic investigation reveals highly conserved, mosaic, recombination events associated with capsular switching among invasive *Neisseria meningitidis* Serogroup W sequence type (ST)-11 strains. Genome Biol Evol.

[15] Lucidarme J, Lekshmi A, Parikh SR, Bray JE, Hill DM, Bratcher HB, Gray SJ, Carr AD, Jolley KA, Findlow J, et al. Frequent capsule switching in 'ultra-virulent' meningococci - are we ready for a serogroup B ST-11 complex outbreak? J Inf Secur. 2017;75(2):95–103. 10.1016/j.jinf.2017.05.015 PMC5522521 28579305

[16] Zhu B, Yao P, Zhang L, Gao Y, Xu L, Xie N, Shao Z. Genetic analysis of *Neisseria meningitidis* sequence type 7 Serogroup X originating from Serogroup a. Infect Immun. 2017;85(6):e01019–16. 10.1128/IAI.01019-16 PMC5442631 28320835

[17] Pan J, Yao P, Zhang H, Sun X, He H, Xie S (2014). The case of a new sequence type 7 serogroup X *Neisseria meningitidis* infection in China: may capsular switching change serogroup profile?. Int J Infect Dis.

[18] Leimkugel J, Hodgson A, Forgor AA, Pfluger V, Dangy JP, Smith T, Achtman M, Gagneux S, Pluschke G (2007). Clonal waves of Neisseria colonisation and disease in the African meningitis belt: eight- year longitudinal study in northern Ghana. PLoS Med.

[19] Lamelas A, Harris SR, Roltgen K, Dangy JP, Hauser J, Kingsley RA, Connor TR, Sie A, Hodgson A, Dougan G (2014). Emergence of a new epidemic *Neisseria meningitidis* serogroup a clone in the African meningitis belt: high-resolution picture of genomic changes that mediate immune evasion. MBio.

[20] Huber CA, Pfluger V, Hamid AW, Forgor AA, Hodgson A, Sie A, Junghanss T, Pluschke G (2013). Lack of antigenic diversification of major outer membrane proteins during clonal waves of *Neisseria meningitidis* serogroup a colonization and disease. Pathog Dis.

[21] Retchless AC, Hu F, Ouedraogo AS, Diarra S, Knipe K, Sheth M, Rowe LA, Sangare L, Ky Ba A, Ouangraoua S (2016). The establishment and diversification of epidemic-associated Serogroup W Meningococcus in the African Meningitis Belt, 1994 to 2012. mSphere.

[22] Agnememel A, Hong E, Giorgini D, Nunez-Samudio V, Deghmane AE, Taha MK (2016). *Neisseria meningitidis* Serogroup X in sub-Saharan Africa. Emerg Infect Dis.

[23] Lucidarme J, Hill DM, Bratcher HB, Gray SJ, du Plessis M, Tsang RS, Vazquez JA, Taha MK, Ceyhan M, Efron AM (2015). Genomic resolution of an aggressive, widespread, diverse and expanding meningococcal serogroup B, C and W lineage. J Inf Secur.

[24] World Health Organization (2011). Laboratory methods for the diagnosis of meningitis caused by *Neisseria meningitidis*, *Streptococcus pneumoniae*, and *Haemophilus influenzae*.

[25] Retchless AC, Kretz CB, Chang HY, Bazan JA, Abrams AJ, Norris Turner A, Jenkins LT, Trees DL, Tzeng YL, Stephens DS (2018). Expansion of a urethritis-associated *Neisseria meningitidis* clade in the United States with concurrent acquisition of N gonorrhoeae alleles. BMC Genomics.

[26] Jolley KA, Brehony C, Maiden MC (2007). Molecular typing of meningococci: recommendations for target choice and nomenclature. FEMS Microbiol Rev.

[27] Wang X, Cohn A, Comanducci M, Andrew L, Zhao X, MacNeil JR, Schmink S, Muzzi A, Bambini S, Rappuoli R (2011). Prevalence and genetic diversity of candidate vaccine antigens among invasive *Neisseria meningitidis* isolates in the United States. Vaccine.

[28] Bambini S, De Chiara M, Muzzi A, Mora M, Lucidarme J, Brehony C, Borrow R, Masignani V, Comanducci M, Maiden MC (2014). Neisseria adhesin a variation and revised nomenclature scheme. Clin Vaccine Immunol.

[29] Stamatakis A (2014). RAxML version 8: a tool for phylogenetic analysis and post-analysis of large phylogenies. Bioinformatics.

[30] Gardner SN, Slezak T, Hall BG (2015). kSNP3.0: SNP detection and phylogenetic analysis of genomes without genome alignment or reference genome. Bioinformatics.

[31] Darling AE, Mau B, Perna NT (2010). progressiveMauve: multiple genome alignment with gene gain, loss and rearrangement. PLoS One.

[32] Guindon S, Dufayard JF, Lefort V, Anisimova M, Hordijk W, Gascuel O (2010). New algorithms and methods to estimate maximum-likelihood phylogenies: assessing the performance of PhyML 3.0. Syst Biol.

[33] Didelot X, Wilson DJ (2015). ClonalFrameML: efficient inference of recombination in whole bacterial genomes. PLoS Comput Biol.

[34] To TH, Jung M, Lycett S, Gascuel O (2016). Fast dating using least-squares criteria and algorithms. Syst Biol.

[35] Letunic I, Bork P (2016). Interactive tree of life (iTOL) v3: an online tool for the display and annotation of phylogenetic and other trees. Nucleic Acids Res.

[36] Cock PJ, Antao T, Chang JT, Chapman BA, Cox CJ, Dalke A, Friedberg I, Hamelryck T, Kauff F, Wilczynski B (2009). Biopython: freely available Python tools for computational molecular biology and bioinformatics. Bioinformatics.

[37] World Health Organization (2013). Meningococcal disease in countries of the African meningitis belt, 2012 - emerging needs and future perspectives. Wkly Epidemiol Rec.

[38] Caugant DA, Kristiansen PA, Wang X, Mayer LW, Taha MK, Ouedraogo R, Kandolo D, Bougoudogo F, Sow S, Bonte L (2012). Molecular characterization of invasive meningococcal isolates from countries in the African meningitis belt before introduction of a serogroup a conjugate vaccine. PLoS One.

