# Supplementary figures and images for: Molecular characterization of invasive meningococcal isolates in Burkina Faso as the relative importance of serogroups X and W increases, 2008–2012
Source: BMC Infect Dis. 2018 Jul 18;18:337. doi: 10.1186/s12879-018-3247-x (PMC6052536; doi:10.1186/s12879-018-3247-x)

# Isolate markers (CC11 NmW)

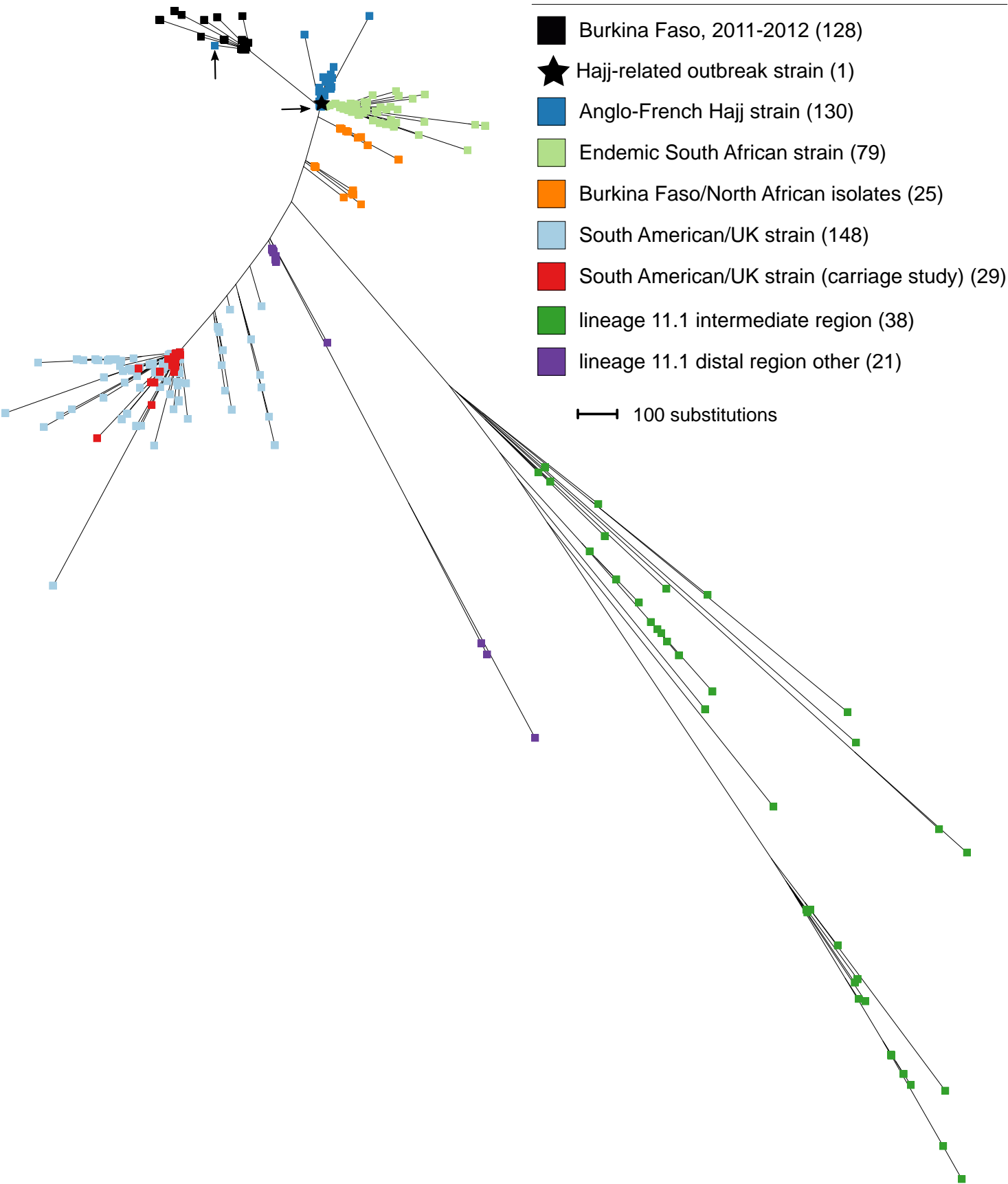

Supplement: Supplementary file 1 — (PDF) Unrooted phylogeny of international NmW CC11 isolates. The 128 isolates from this study are identified by black squares, the Hajj-related outbreak isolate is identified by a black star, and the remaining 470 isolates are identified according to the categories defined by from Lucidarme et al. [23]. Arrows mark isolates that are not in the same category as the most closely related isolates: the Hajj-related outbreak isolate (M07149) and an “Anglo/French Hajj strain” isolate collected in France during 2014 (M14 240,446). The tree is scaled by the number of parsimonious substitutions per branch, identified by kSNP3. Branches with bootstrap support < 70% have been deleted. (PDF 33 kb) [file 12879_2018_3247_MOESM1_ESM.pdf]
